# Enhancing Care Coordination in Oncology and Nononcology Thoracic Surgery Care Pathways Through a Digital Health Solution: Mixed Methods Study

**DOI:** 10.2196/60222

**Published:** 2024-11-26

**Authors:** Véronique Nabelsi, Véronique Plouffe

**Affiliations:** 1 Département des sciences administratives Université du Québec en Outaouais Gatineau, QC Canada; 2 Département des sciences comptables Université du Québec en Outaouais Gatineau, QC Canada

**Keywords:** digital health solution, care coordination, optimization, health care providers, oncology, nononcology, thoracic surgery, pathways, continuity of care, interfacility, Quebec

## Abstract

**Background:**

Health-system fragmentation in Quebec significantly impacts care coordination, leading to interruptions in patients’ care pathways and adverse effects on their health. Coordinating interfacility service corridors is complex and requires collaboration between multiple health care providers (HCPs) and care settings. Effective care coordination is essential to ensure optimal patient management at transition points.

**Objective:**

This study aims to improve oncology and nononcology thoracic surgery care pathways by enhancing care coordination during interfacility transfers through a digital health solution.

**Methods:**

A multicenter implementation study was conducted across 2 health regions and 2 health care facilities in Quebec. We conducted 27 semistructured interviews with HCPs and managers to better understand the care pathways. Participatory design workshops were held with future users and key stakeholders at an early stage of the technology’s design to validate the prototype’s functionalities and workflows. A web survey was sent to all end users (N=13) to assess their experience with the platform.

**Results:**

All participants (100%) either “agreed” or “strongly agreed” that the platform provided significant benefits. It enhanced interestablishment coordination (4/13, 31% agreed and 9/13, 69% strongly agreed) and continuity of care and services (8/13, 62% agreed and 5/13, 38% strongly agreed), and it contributed to better management and patient intake (10/13, 77% agreed and 3/13, 23% strongly agreed) and process fluidity (3/13, 77% agreed and 3/13, 23% strongly agreed). Surgeons from the McGill University Health Centre confirmed that the platform facilitated and secured information transmission (2/5, 40% agreed and 3/5, 60% strongly agreed) and kept track of oncology patient referrals, follow-up needs, and cases where surgery is unnecessary (2/5, 40% agreed and 3/5, 60% strongly agreed). Nursing staff from the Centre intégré de santé et de services sociaux de l’Outaouais and McGill University Health Centre reported high satisfaction with the platform’s support during preoperative visit, surgery, and discharge processes. All participants perceived the platform as intuitive and easy to use. Additionally, participants valued its efficiency in providing rapid access to patient data, which reduces task time and ensures document security, thereby improving care coordination across facilities. The project’s success has convinced the HCPs and senior management at both health care facilities to pursue long-term use of the Akinox digital health platform.

**Conclusions:**

This pilot project represents a significant advancement in thoracic surgery care pathways and the coordination of interfacility health care service corridors. The project provides care pathways that are adaptable to other surgical specialties. It also paves the way for improving care in cancer and other health care networks while highlighting the key role of nurse navigators in patient care management. The project underscores the value of strategic leadership and stakeholders’ collaboration to improve care coordination and operational efficiency by demonstrating technology’s essential role in patient care pathways.

## Introduction

### Background

In Quebec, as in other provinces in Canada, care coordination is an important issue due to the fragmentation of the health system, which is also observed around the world [1-4]. A key issue is the lack of coordination between health care providers (HCPs), which can lead to interruptions in the patient’s care pathway, adversely affecting their health and well-being [5-7]. The thoracic surgery care pathways are complex, requiring collaboration between various HCPs from different disciplines and settings [8-10]. The coordination of interfacility service corridors is even more complex for certain types of specialized health care, such as thoracic oncology surgery [11-13], where patients may require surgery, radiotherapy, or chemotherapy [14]. Effective care coordination ensures safe and efficient care transitions, promoting patient safety and care quality [15-18].

Care transitions involve different HCPs, requiring multidisciplinary communication, coordination, planning, and shared accountability [19,20]. Effective care coordination involves the timely exchange of concise, complete, and relevant information between different HCPs within the same health care facility or from one facility to another, to ensure patient care management at transition points [18,21-27]. However, transitions between facilities are critical points where continuity may be compromised if there is a lack of coordination [28-34].

Studies have shown that a lack of care coordination reduces system performance and negatively impacts patients’ health and quality of life [35-41]. Susceptible patients or those with complex needs are particularly affected [42,43]. Poor coordination leads to duplicated tests or treatments, medical errors, increased costs, and mismanaged transitions, all of which comprise patient satisfaction and care quality [13,25,44-49]. By contrast, effective coordination reduces emergency room visits, hospital readmissions [26,35,50,51], delays, and adverse events [35,52,53].

### Digital Technologies and Care Coordination

It is well known that the computerization of the Quebec health care network, and clinical computerization in particular, lags behind that of other Canadian provinces, contributing to fragmented patient records [54-56]. However, this issue is also observed worldwide. For example, patients’ medical information is scattered across different systems and not easily accessible or shared between HCPs [57,58]. Overall, the flow of information, both within and between health care facilities, is deficient, and patients constantly have to repeat their information or undergo unnecessary tests or examinations simply because the information is inaccessible [59-64].

Information and communication technologies (ICTs) are increasingly perceived as tools that can improve the quality of care, patient safety, and the efficiency of the health care system [65-71]. ICTs provide HCPs with real-time access to patient information, eliminating redundant or unnecessary tests and procedures [72-78], and facilitating multidisciplinary collaboration [72-82]. In addition, ICTs enhance care traceability [70] and promote evidence-based medicine [83]. Automating administrative tasks through ICTs can optimize resource use and improve patient satisfaction [76,84-88].

To ensure efficient coordination of interfacility thoracic surgery care and services, it is important that the health care facilities work together in a transparent and coordinated way, sharing patient information and ensuring seamless continuity of care. Digital health solutions are required to make workflows more efficient and ensure that patients receive the right care at the right time [76,89,90]. Therefore, this study aims to improve oncology and nononcology thoracic surgery care pathways by enhancing care coordination by first analyzing the interfacility process, then designing, adapting, and testing a customized digital platform and finally implementing the solution while assessing the end-user experience.

## Methods

### Ethical Considerations

Ethics approval was obtained from the research ethics committee of the Centre intégré de santé et de services sociaux de l’Outaouais (CISSSO) before the beginning of the study (2019-258_141_MP), in Quebec. All participants provided written informed consent before participation. The privacy rights of the study participants were observed. The study participants did not receive monetary compensation.

### Pilot Project Context

The pilot project focused on the provision of interregional services between 2 facilities, namely the McGill University Health Centre (MUHC) in Montreal and the CISSSO in Gatineau. This service corridor enables the efficient use of the Community Health and Social Services Network’s resources so that patients’ needs can be met as quickly as possible.

In 2014, Quebec’s Ministère de la Santé et des Services sociaux (MSSS) approached the MUHC, which has a supraregional team of experts, to establish a close collaboration with affiliated centers specializing in lung and esophageal cancer cases. The CISSSO has an exclusive thoracic surgery service corridor with the MUHC. As part of this collaboration, MUHC surgeons spend 3 to 4 days per month at the CISSSO for clinic visits with >50 patients per clinic, and the surgeons perform >200 surgical procedures on these patients at the MUHC each year. It should be noted that there are >1000 consultations at the CISSSO every year.

In September 2018, the Direction générale de cancérologie reconfirmed the added value of such networking and mandated the MUHC to create a pulmonary oncology network to optimize care pathways and service corridors with its affiliated centers. In addition, the 2015 to 2025 National Public Health Program produced by Quebec’s MSSS emphasizes the importance of organizing health care in a way that will ensure the continuity of health care services, better harmonize transitions at different levels of the health care system, and avoid duplication of services. A key element of this structure is facilitating accessibility and coordination between health care units within the same region to ensure the complementary nature of their service offering and between regions when specialized services are required.

The oncology and nononcology thoracic surgery pathways, where the referral center (MUHC) and the affiliated center (CISSSO) must collaborate on a series of clinical and administrative activities, have a critical mass of patients that is very well suited to a pilot project that can be replicated in other specialties. Specifically, the care pathways involve a complex organization of resources and patient flows. There are many reasons to optimize the care pathways. First, coordination between health care facilities has become risky. In addition, the process of transmitting patients’ clinical information, the administrative documents relating to this information, and the monitoring of the continuum of services are unstable and insecure. A technological solution can play an important role in bridging these gaps.

Following initial analyses of clinical and administrative flows, the need to optimize the care pathways became clear, with a focus on improving safety, and facilitating care coordination through the implementation of an integrated digital health solution. The three main and interdependent objectives of this implementation are (1) to understand the interfacility thoracic surgery pathways; (2) to design, adapt, and test the platform with the target pathways; and (3) to implement the platform and evaluate the end-user experience.

### Study Design and Settings

#### Overview

A pathway refers to a care plan that details the specific steps for managing the care of a patient with a specific pathology to ensure high-quality, consistent, and continuous care [91]. For this purpose, this study used an integrated knowledge mobilization approach, a partner-centered approach that seeks to improve outcomes by involving all relevant partners (political decision makers, managers, HCPs, community members, patients, digital health professionals, etc) throughout the research process [92-94]. This approach theorizes that the coconstruction of knowledge is likely to result in relevant, applicable, and transferable knowledge for end users [73,92-97].

We also used an exploratory mixed methods approach that combines different sources of data to determine and respond to the needs of HCPs and to improve care coordination that will enable continuous and consistent management of patients throughout the care pathways. To meet the 3 main, interdependent objectives, we conducted a multicenter implementation study at 2 health care facilities (MUHC and CISSSO) located in 2 different health regions (Montreal and Gatineau) in the province of Quebec. For the first 2 objectives, we used qualitative research methods, while the third objective involved a quantitative evaluation. The COREQ (Consolidated Criteria for Reporting Qualitative Research) [98] checklist was used to ensure that the study met the recommended standards of qualitative data reporting (Multimedia Appendix 1).

#### Data Collection and Analysis of Objectives

##### Objective 1: Understand the Interfacility Thoracic Surgery Care Pathways

To facilitate the achievement of objective 1 and ensure the collection of the greatest possible amount of quality information, an interview guide was created and used at both health care facilities. Each respondent was given a copy of the guide in advance, ensuring they were informed of the covered topics. The aim of the guide was to help gain a better understanding of the interfacility thoracic surgery care pathways related to 3 main components. The first component was the context of clinical and administrative flows. The second component encompassed the clinical and administrative forms and official documents used throughout the care pathways. The third component included the major problems encountered by HCPs.

Sampling was purposive, given the 2 chosen health care facilities and the different types of target participants (thoracic surgeons, nurses, oncology nurse navigators, managers, and medical secretaries). All participants (N=27) were contacted by email, which included the interview guide and consent form. The form explained the context, project objective, procedure and duration, anticipated benefits, as well as anonymity and confidentiality. Everyone agreed to take part in the project. Free and informed consent was obtained from all participants at the scheduled data collection meetings. A total of 27 semistructured individual face-to-face interviews, each lasting 60 minutes, were conducted over a 6-month period between June and November 2019 at the MUHC and the CISSSO by the researcher. All interviews were audio recorded with participants’ permission. The participants did not have any personal or professional relationship with anyone from the research team.

The interviews were transcribed by the researcher in Microsoft Word. The interviews were analyzed in isolation to highlight the experiences and concerns associated with the pathways. A summary of the analysis of each interview was validated with the respondents.

##### Objective 2: Design, Adapt, and Test the Platform With the Target Pathway

To facilitate the achievement of objective 2, which consists of designing, adapting, and testing the digital health platform with the target care pathways, we used the participatory design approach. This approach, also known as cocreation or end user–centered design, is advocated to foster the development of health care technology solutions [99,100]. Numerous studies illustrate the benefits of incorporating the perspectives and knowledge of future users at the outset of the technology design and development process [101-104].

As part of this pilot project, a close collaboration was established with doctors, nurses, medical secretaries, researchers, developers, designers, and other key stakeholders. Various qualitative methods were used to support stakeholder involvement.

In the first stage, based on the interviews conducted face-to-face under objective 1, we were able to (1) identify the clinical and administrative needs of future users and (2) map the current thoracic surgery care pathways to identify optimization opportunities and implement targeted improvements. This information was gathered from the following 13 participants: 5 (38%) MUHC surgeons, 3 (23%) CISSSO nurses, 3 (23%) MUHC nurses, and 2 (15%) MUHC medical secretaries.

During the second stage, based on the previous results, we mapped the target interfacility thoracic surgery pathways. Our objectives were to identify the key steps of the care pathways and to illustrate how the platform can help improve care coordination and management at each step. We carried out an analysis of existing workflows using the Business Process Model Notation method with Visio software 2021 (Microsoft Corporation). All (13/13, 100%) participants mentioned earlier were involved in validating the map of the current care pathways.

On the basis of the data generated by the first 2 stages, we organized web participatory design workshops over an 18-month period, with the aim of actively involving future users in the technology design process. Each workshop, conducted via Microsoft Teams teleconference, lasted 90 minutes and was visually recorded with participants’ permission. In addition, the researcher took notes throughout the sessions. A total of 11 participants took part in the workshops, including 1 (9%) researcher, 3 (27%) developers and designers, 2 (18%) surgeons, 2 (18%) nurses, 1 (9%) manager, 1 (9%) Réseau universitaire intégré de santé et de services sociaux McGill Telehealth Coordination Centre consultant, and 1 (9%) MUHC clinical coordinator. These workshops generated knowledge coproduced with the participants, which was incorporated into the prototype under development.

On the basis of the data generated during the participatory design workshops, a prototype was designed and discussed with end users and other key stakeholders during feedback meetings. The aim was to validate certain functionalities and workflows that were planned for the prototype. Subsequently, the Akinox team carried out an iterative review of the prototype following its usual development process. Akinox is a company that develops digital solutions for health care organizations and was our technology partner for this pilot project.

By the end of this process, the requirements for the adaptation and finalization of the Akinox digital health platform were established. This enabled us to prepare the platform for implementation in the target setting, considering the feedback from future users and ensuring that the design meets their needs and preferences. This iterative, user-centered approach resulted in a final product that is better adapted and more user-friendly for end users.

##### Objective 3: Implement the Platform and Evaluate the End-User Experience

The Akinox digital health platform was rolled out in January 2021. Each region was provided with cloud-based access to the platform as agreed in collaboration with IT units in each health region. We used a phased approach to implementation. Training sessions were organized in each setting, and a user guide was sent to the 13 end users of the platform.

In December 2021, we conducted a web-based survey of all MUHC and CISSSO HCPs (N=13) involved in the thoracic surgery care pathways to assess their experience of using the platform. All participants received an email with a link to the web-based survey, which was conducted using SurveyMonkey software (SurveyMonkey Inc), sent by the researcher.

The survey comprised four parts: (1) demographic information (region of origin, health care facility, and profession), (2) perceived benefits of the platform, (3) assessment of the platform in terms of specific workflows for each user profile, and (4) assessment of the overall user experience of the platform.

For part 2, respondents rated the perceived benefits of the platform using a 5-point Likert scale ranging from “strongly disagree” to “strongly agree.” Questions focused on the positive aspects of the platform that improve work efficiency, care coordination, etc. In part 3, respondents rated the suitability of the platform for their specific tasks using a 5-point Likert scale. For part 4, respondents were asked to answer open-ended questions focusing on the factors of acceptance and use of the platform. The open-ended questions focused on ease of use, user-friendliness, expected effort, expected performance, perceived usefulness, etc.

## Results

### Objective 1: Understand the Interfacility Thoracic Surgery Care Pathways

#### Overview

The existing oncology and nononcology thoracic surgery care pathways include the reference center (MUHC) and the affiliated center (CISSSO). Under a formal agreement, the 2 health care facilities must collaborate on a series of clinical and administrative activities in support of patients with lung and esophageal cancer, with the aim of providing patients with a seamless care experience. The MUHC is recognized for its leading-edge expertise and has a supraregional team dedicated to the treatment of lung and esophageal cancer. The CISSSO has a designated interdisciplinary team to ensure complementarity and continuity in the care and services provided to oncology (lung and esophageal cancer) and nononcology patients.

#### Context of Clinical and Administrative Workflows

**Figure 1:**
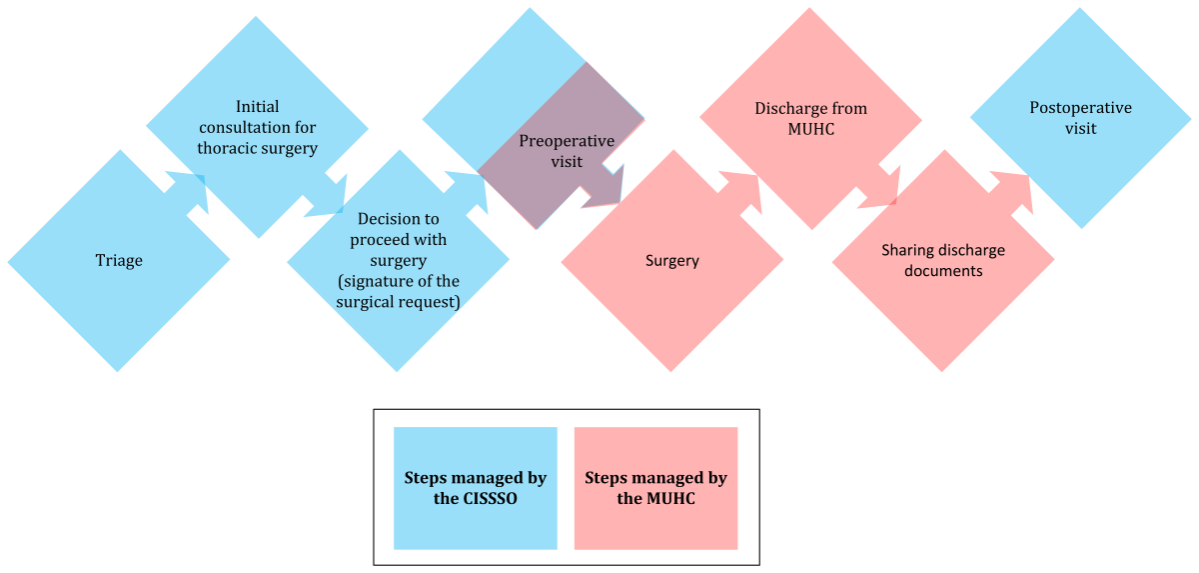
Thoracic surgery patient care pathways at the McGill University Health Centre (MUHC) and the Centre intégré de santé et de services sociaux de l’Outaouais (CISSSO). Steps in blue represent those managed by the CISSSO, while steps in red indicate those managed by the MUHC.

Figure 1 provides a macro view of the thoracic surgery process. It begins with the receipt of a request for a patient consultation with a surgeon and ends with the patient being discharged from the hospital after surgery.

Patient care management involves a large number of human resources. Coordination between HCPs in the same facility and between health care facilities is essential to ensure that patient care management is carried out according to priority and pathology. The oncology and nononcology care pathways are complex in terms of scheduling patients for timely treatment in a variety of care settings.

At the CISSSO, there are 2 care settings: the outpatient clinic and the oncology clinic. The outpatient clinic provides consultation and follow-up services to patients. These may include follow-ups to examinations requiring surgery, pre and postsurgery care, as well as pre and posthospitalization care for general surgery and biopsies. The oncology clinic cares for patients with cancer. Each clinic has assembled a care team to educate patients about their disease; provide the care and services required by their condition; deliver education related to their condition; and provide support to patients, their families, and loved ones throughout the care pathway.

At the MUHC, there are 4 care settings: the thoracic surgery clinic, the preoperative clinic, the care unit, and the oncology clinic for certain patients. The thoracic surgery clinic provides consultation, postexamination, postoperative, and posthospitalization follow-up services. It specializes in the investigation and treatment of potential or diagnosed thoracic pathologies (lung cancer, esophageal cancer, etc). The preoperative clinic conducts the patient’s preoperative health check. During this visit, patients undergo various medical examinations. The visit is also used to schedule the hospital stay and discharge following the operation. The care unit is an inpatient unit that receives patients arriving from the operating room. The MUHC oncology clinic provides the same services as the CISSSO.

This service organization model involves an agreement between the MUHC and the CISSSO to deliver the service offering and meet the needs of the Gatineau region’s population. CISSSO patients must travel to another health care facility (MUHC) in another region (Montreal) to receive the care and services they need. Patients must travel more than 200 km from Gatineau to Montreal.

The oncology and nononcology thoracic surgery patient care pathways are complex, involving several transitions with HCPs at scheduled times and in specific care settings.

#### Clinical Forms and Other Documents

In today’s thoracic surgery pathways, it is common to have several forms to complete at different times (preoperative forms, forms related to the surgery, and postoperative forms) and health care facilities (MUHC and CISSSO) and by various HCPs. These forms and documents are used to collect essential information to ensure appropriate patient management and effective communication between members of the care team at each health care facility.

During the interviews, we identified 25 forms, such as the request for admission to surgery, the blood transfusion consent form, and the informed consent form. In addition to the forms, other documents formed part of the patient’s medical record, such as clinical notes filed by consultation date, pathology and imaging reports, laboratory results, and the discharge summary after a hospital stay. We also listed 13 pre- and postoperative guides or instructions provided to patients. These documents were designed to inform patients about the various stages of their journey, prepare them for surgery, and help them recover effectively after the operation.

All documentation is kept in the patient’s file in paper form at both the CISSSO and the MUHC. In addition, surgeons pick up these documents and physically transport them to the health care facility, where the next stage of treatment or care management will take place.

#### Major Problems in the Current Care Pathways

##### Overview

Analysis of the pathways illustrated major dysfunctions attributable to the fact that the patient’s care pathway is shared between 2 health care facilities operating in silos. Organizational silos have a negative impact on integration, as each entity focuses on its own area of responsibility at the expense of improving efficiency. Through the interviews, we identified 4 areas that explain the coordination issues in the interfacility thoracic surgery service corridors.

##### Communication and Management and Transmission of Information

The management and transmission of information between the MUHC and the CISSSO are deficient and present an increased risk of error. Lack of communication between the silos further complicates the situation, to the point where information is sometimes missing, incomplete, or processed twice. The CISSSO frequently sends the results of preoperative visits twice, by email and by post, to ensure that the MUHC receives them. However, these results arrive at different care units within the MUHC, making it difficult to verify that a patient’s file is complete. When surgeons cannot find the necessary tests, they are forced to repeat them. This causes delays, exposes the patient to potential risk, and impacts the quality of care provided.

In addition, having surgeons physically transport documents between health care facilities presents certain disadvantages and risks. First, there is the risk of documents being lost, damaged, or misplaced during transport. Second, surgeons at the MUHC sometimes forget the documents when they travel to the CISSSO clinic.

The absence of this information impairs the process considerably, given that decision-making throughout the pathways is highly dependent on the availability of certain key pieces of information. Not having these key elements triggers a “scramble for information” that consumes a staggering amount of energy and time. This leads to conflicts between the HCPs who are required to generate the information and those who need it to carry out their clinical and administrative duties properly.

In this particular case, the movement of patient care management information between the CISSSO and the MUHC is not secure and reliable, and this information is not systematically filed in the patient’s clinical record. The process for exchanging patient information between the health care facilities is not harmonized and is sometimes dysfunctional and error-prone.

##### Coordination of Care

Ineffective communication between the different HCPs and between the health care facilities as well as the absence of an integrated information system to facilitate rapid and secure sharing of medical information and patient follow-up appear to impact coordination between the different steps of the care pathways and between HCPs. For example, information such as test results and treatment plans is missing when patients are transferred from one department to another or from one hospital to another. This is also the case for coordination and synchronization between surgeons and nurses, with patients finding it difficult to move seamlessly from one step of the care pathway to another.

Lack of communication makes it difficult to ensure that a patient’s care and services progress smoothly, particularly when it comes to appointment reminders and follow-up after hospitalization or an examination. In fact, the lack of follow-up mechanisms between the different HCPs makes it difficult to follow a patient’s progress along their care pathways, complicating the scheduling of care, including the scheduling of surgery. In the absence of adequate follow-up, some patients may need additional tests before surgery or require chemotherapy before surgery. If these needs are not identified and addressed in time, it can lead to delays in preparatory care and compromise the quality and effectiveness of care. Sometimes patients are examined too late when their cancer is already at an advanced stage due to gaps in follow-up.

Duplication of preoperative patient preparation steps between the CISSSO and the MUHC leads to inefficiencies and additional investigations. Even when the patient has already been cleared for discharge at the CISSSO after a preoperative visit, certain relevant information about their medical condition is not properly documented and transmitted to the MUHC.

Frequent changes in MUHC operating schedules pose major challenges when scheduling the resources needed for each surgical procedure. This includes operating rooms, medical and nursing staff, equipment, and supplies. When schedules change, it is difficult to ensure that all resources are available at the right time, leading to delays and cancellations. Unfortunately, these unexpected changes in dates cause anxiety and uncertainty, especially if they are announced at the last minute. Patients and their families must make rapid adjustments to the organization of their hospital stay and to the support they require. Moreover, the fact that CISSSO patients are required to travel a great distance—more than 200 km—to get to the MUHC adds another challenge.

A common problem is the lack of coordination with primary care providers and hospitals after surgery. For example, there is inadequate coordination of postoperative patient follow-up, including referral and collaboration with community resources, such as local community service centers (LCSCs) or other home care services. There is a paucity of clear guidelines defining responsibilities and referral steps between health care facilities and community resources. Moreover, the lack of formalization leads to confusion about who is responsible for which tasks and results in insufficient coordination of care. This can compromise patient recovery.

##### Continuity of Care and Services

Many issues were raised regarding the continuity of care and services between the 2 health care facilities, namely the absence of certain essential information, such as the patient’s discharge date and the MUHC’s discharge summary. This hinders proper postoperative follow-up and compromises overall patient care management at the CISSSO. The CISSSO nurse must make phone calls to the MUHC to obtain the documents by fax. Furthermore, the CISSSO’s attending physicians do not always receive discharge summaries from the MUHC hospital; these summaries contain essential information on their patients’ health status.

The high volume of calls and patients represents a challenge for their care management and follow-up. To meet this challenge, CISSSO nurses must decide which patients to prioritize every day. There is also a lack of continuity in requests for pathology services, and it takes several days to obtain results.

Patients are not systematically told about their postoperative appointments when they are discharged from the MUHC hospital, and they leave the health care facility without knowing whom to contact for a follow-up appointment, leading to confusion and difficulty in obtaining follow-up care at the CISSSO. The CISSSO is not aware of the specific dates of surgeries and discharges, so it is difficult to schedule and inform patients of postoperative appointments before they leave the MUHC. In fact, it is often the patient who calls the CISSSO to say that they have not been given a postoperative appointment.

Furthermore, the CISSSO frequently receives phone calls from patients who have not received a call from the LCSC to change their dressing or receive other necessary care after surgery. CISSSO patients are not always contacted by the LCSC for their postsurgery care management, and the interfacility service request (IFSR) is sent to the wrong LCSC in and around Gatineau. In fact, the MUHC does not always confirm the patient’s home LCSC before sending the IFSR. Therefore, the MUHC must prepare another IFSR for follow-ups at another LCSC. In addition, the type of required follow-up (home or outpatient care) is often missing from the IFSR. This information is important to ensure that the patient receives the appropriate services according to their postoperative needs and to ensure adequate continuity of care.

##### Defining and Understanding Roles

Some tasks are not uniformly and systematically performed from one health care facility to another. Approaches and practices between HCPs and care settings are not harmonized. These disparities lead to fragmentation of care and loss of efficiency across the thoracic surgery care pathways.

In addition, the oncology and nononcology pathways involve numerous players whose roles are sometimes inadequately defined. There are overlaps, duplications, and gaps in patient care management. The absence of an integrated information system can make coordination more difficult and lead to inefficient processes.

### Objective 2: Design, Adapt, and Test the Platform With the Target Pathways

#### Step 1.1: Clinical and Administrative Needs of Future Users

Future users identified needs related to the workflows and future use of a digital health platform aiming to cover the entire interfacility thoracic surgery care pathways from initial triage when the patient is assessed to patient discharge after surgery.

MUHC and CISSSO HCPs submitted a list of administrative and clinical needs that were classified under the following three categories: (1) communication of administrative, medical, and paramedical information; (2) clinical and organizational practices; and (3) human resources (Textbox 1).

**List of administrative and clinical needs.**

**Elements included by the participants related to the need for effective communication and informational continuity**

- Enable the rapid and secure transmission of information and documents required for the continuity of care and services from one health care provider (HCP) to another within the same facility and between health care facilities
- Ensure confidentiality and data protection when transmitting information
- Ensure easy and direct access, without intermediaries, to patient medical data, such as medical records, test results, and x-ray results
- Ensure automatic updating of information and documents without duplication
- Promote the use of standardized and harmonized documents and forms between the McGill University Health Centre and the Centre intégré de santé et de services sociaux de l’Outaouais
- Implement a centralized dashboard to share information in real time
- Promote the harmonization and standardization of communication between HCPs within the same facility and between health care facilities
- Promote the interoperability of interfacility IT systems

**Other specific needs raised by the participants in terms of clinical and organizational practices**

- Enable real-time tracking of the steps in the patient’s care pathways
- Enable efficient management of tasks and flows, with reminders and notifications
- Implement processes to achieve and maintain ministry targets for thoracic surgery
- Implement measures to reduce wait times for diagnostic examinations and preoperative tests
- Establish standardized protocols for preoperative investigations
- Implement mechanisms to limit changes in surgery dates
- Implement standardized procedures for patient follow-up after thoracic surgery, including postoperative visits

**Human resources-related needs identified as part of the implementation of the platform**

- Clarify and define the roles and responsibilities of each HCP within the care pathways
- Propose guidelines for the organization of care and services
- Facilitate patient access to care teams by establishing clear communication channels and optimizing appointment scheduling processes
- Implement a change management plan to support HCPs in adopting the platform

#### Step 1.2: Targeted Improvements

Following the mapping of the current thoracic surgery care pathways, several targeted improvements were implemented. The first improvement was a review of the roles and responsibilities of each HCP involved in the pathways. This helped clarify the responsibilities of each member of the care team to ensure effective coordination and optimal patient care management.

The second improvement was the recruitment of a nurse navigator at both the MUHC and the CISSSO. This person plays the role of monitor, ensuring continuity of care and services throughout the patient’s pathway. She acts as a liaison between the patient and all the HCPs, facilitating communication and enabling more fluid, personalized care management.

The third improvement was the revision of organizational processes surrounding the coordination of activities relating to medical records and the storage of documents in the patient’s file. This revision has improved efficiency and prevented delays or errors in the management and tracking of patient medical data.

The fourth was the rationalization of clinical and administrative flows between health care facilities. This approach has optimized the processes, ensuring smooth, efficient care management throughout the care pathways.

Finally, the last improvement was demonstrating the relevance of a platform for interfacility care management of patients who underwent thoracic surgery. Key aspects of this demonstration included a secure environment for sharing patient medical information, automated notifications to HCPs at different steps of the patient’s care pathway, and the assignment of different user profiles (eg, surgeons and nurses) according to their roles and responsibilities in the care pathways.

#### Step 2: Key Phases of the Target Pathways

The key steps of the target care pathways for interfacility thoracic surgery are triage of the consultation request (CISSSO), preparation of the surgical consultation (CISSSO), consultation (CISSSO), patient referral to surgery, preparation of the preoperative visit (MUHC or CISSSO), the outcome of the preoperative visit (MUHC or CISSSO), surgery (MUHC), hospitalization (MUHC), patient discharge and referral (MUHC), and sharing postdischarge documents (MUHC). These different phases are characterized by round-the-clock user access to clinical information and documents. The key steps, from triage of the consultation request to facilitating access to clinical documents, aim to improve coordination, quality of care, and the accessibility of information throughout the patient’s care pathway.

**Figure 2:**
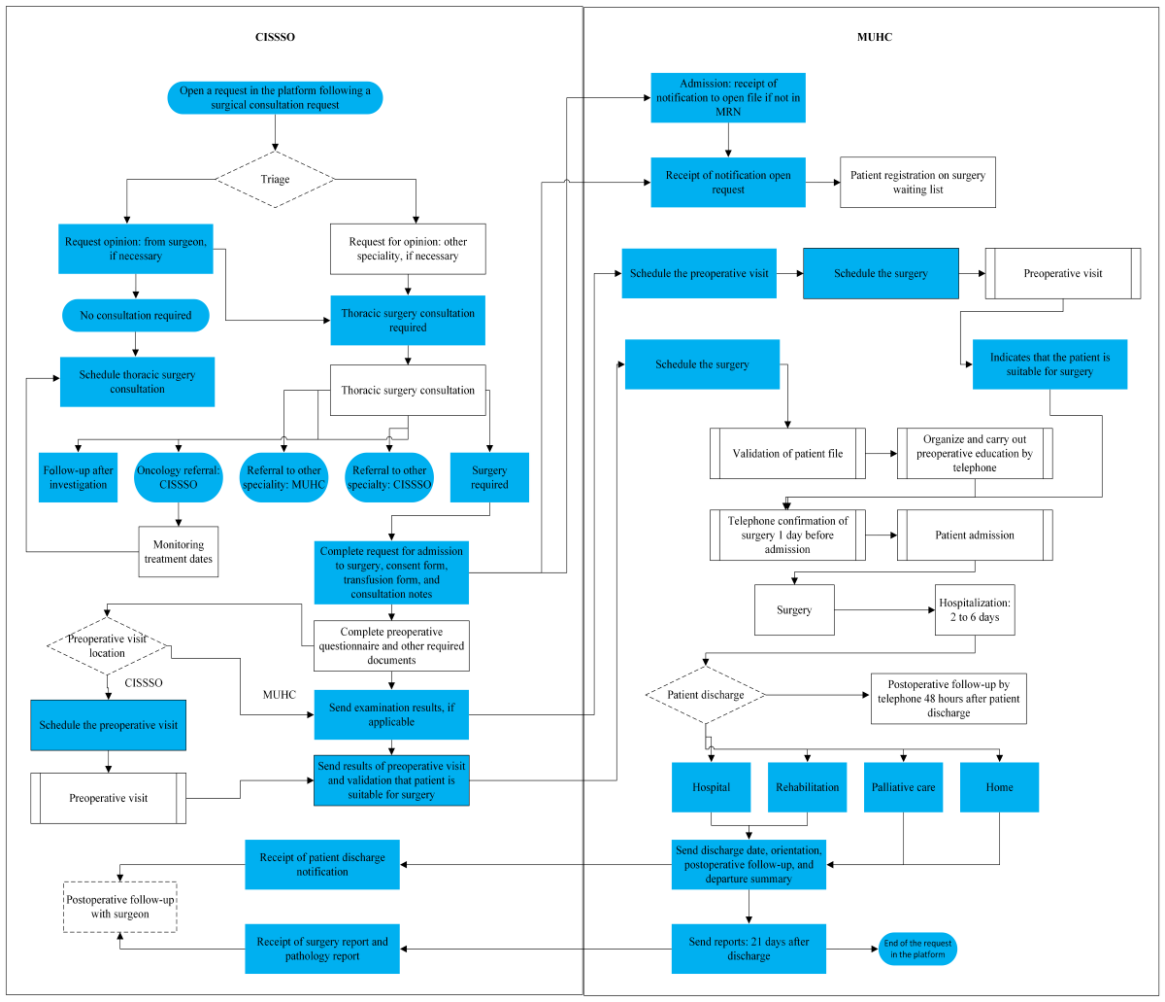
Target interfacility thoracic surgery care pathways integrating the platform. Cells in blue are the actions and information transmitted by the platform for each facility. CISSSO: Centre intégré de santé et de services sociaux de l’Outaouais; MRN: medical record number; MUHC: McGill University Health Centre.

The target pathways illustrated in Figure 2 integrate the platform as well as the CISSSO and MUHC interfaces.

By integrating the Akinox digital health platform and optimizing care coordination processes, the target care pathways aim to provide seamless, coordinated patient care (Figure 2).

Textbox 2 shows the different modules of the platform.

**Modules and functionalities of the Akinox digital health platform.**

**Triage**

- Patient registration (creating a request and selecting a patient)
- Initial triage (surgery consultation not required and surgery consultation to be scheduled)
- Result of the triage

**Schedule the thoracic surgery consultation**

- Book appointment

**Thoracic surgery consultation**

- Consultation (consultation date and results)
- Admission to surgery (3 electronic forms: admission and surgery form, consent form, and transfusion consent form)

**Preoperative scheduling and execution**

- Schedule the preoperative visit
- Preoperative visit

**Patient care management and discharge**

- Schedule the surgery
- Education by telephone
- Documents and examinations
- Patient discharge
- Postdischarge reports

**Figure 3:**
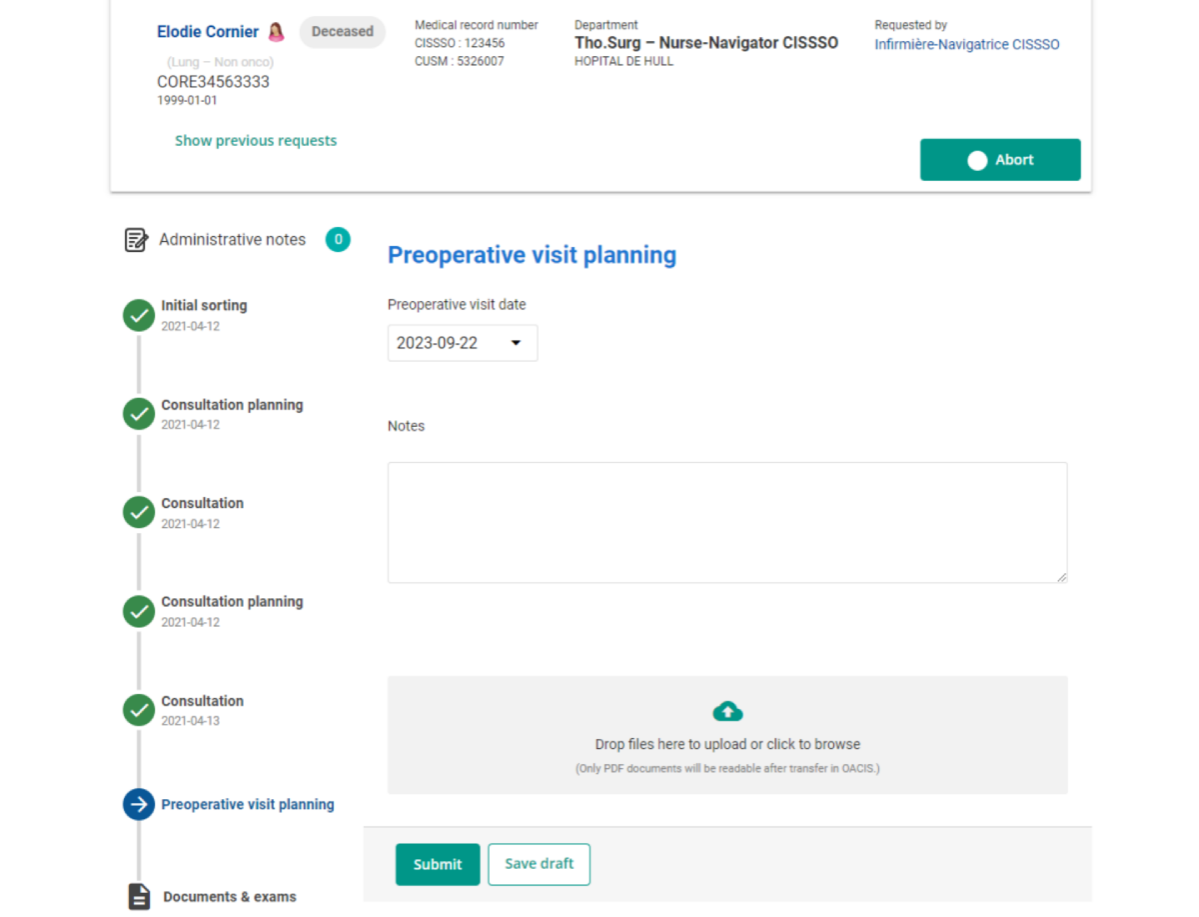
"Metro line"—flowchart for scheduling the preoperative visit. Disclaimer: The patient described in this figure is fictitious.

**Figure 4:**
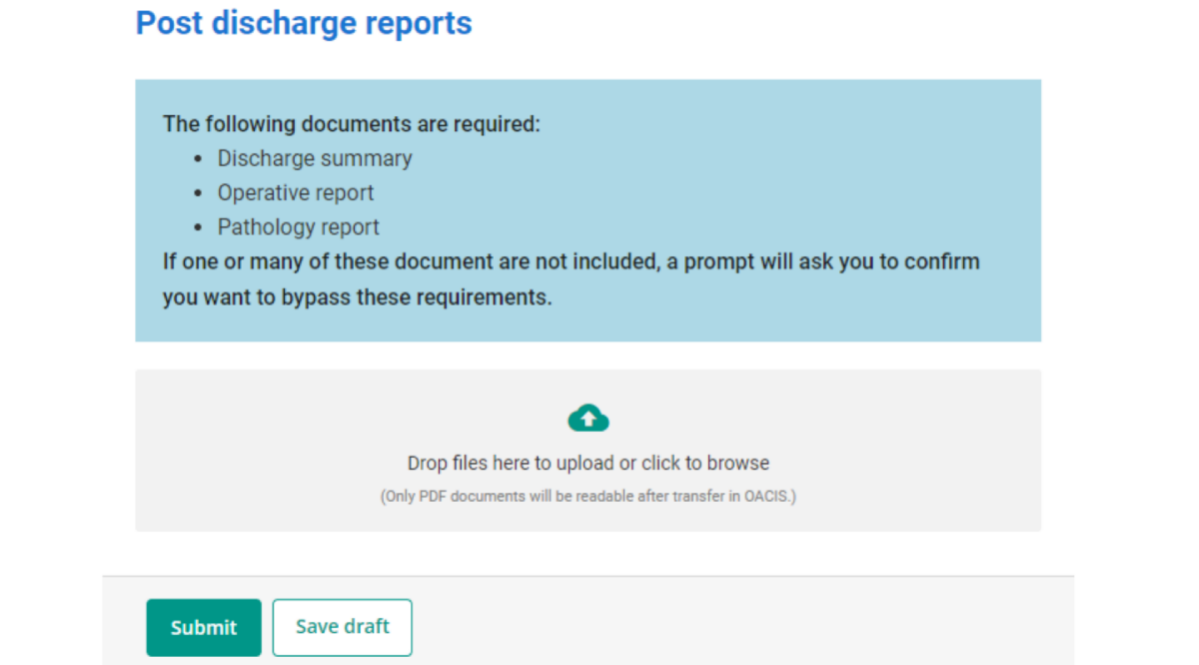
Submitting postdischarge reports.

**Figure 5:**
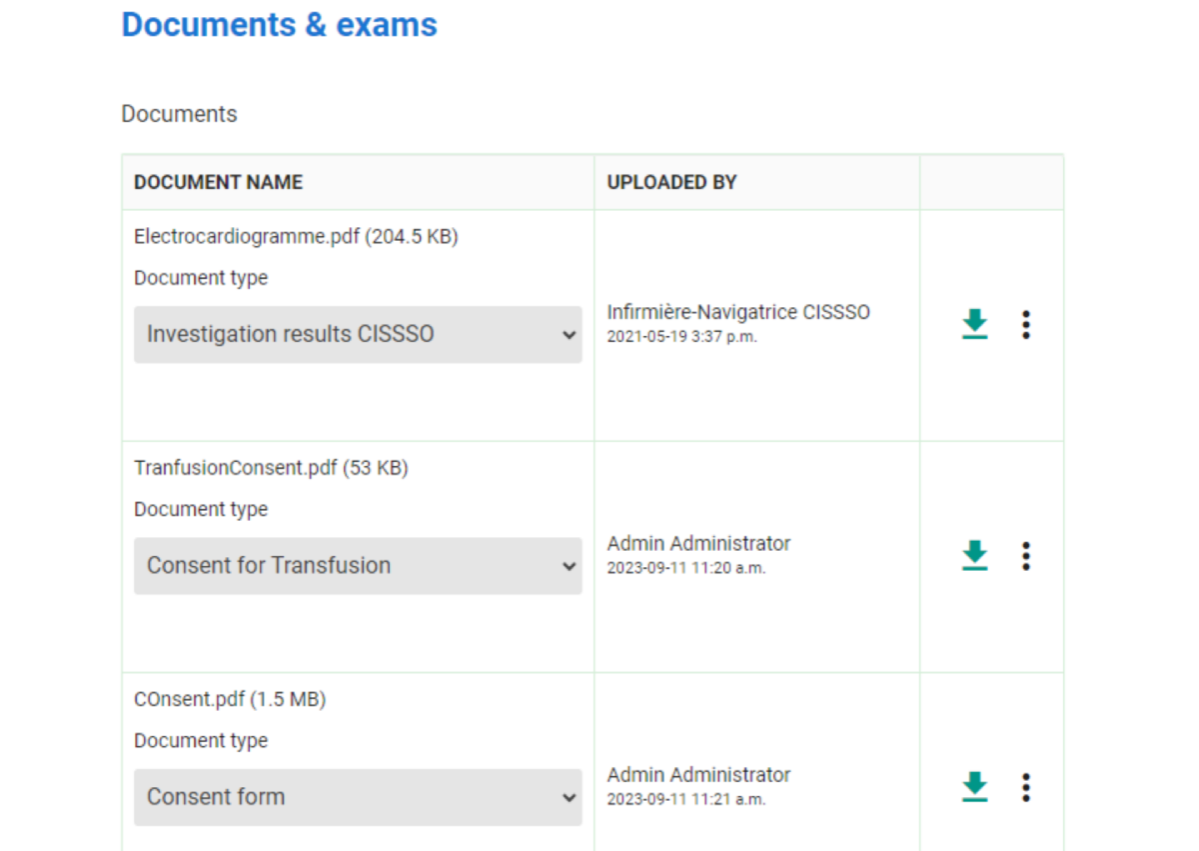
Documents and Examinations section of the platform.

Figures 3-5 present screenshots of the Akinox digital health platform. The platform makes it possible to track each key step in the care pathways. Specifically, the “metro line” (ie, the platform’s term for “flowchart,” shown on the left side of Figure 3) provides a visual overview of the patient’s progress along the care pathways, indicating their current location in the overall process. This facilitates the coordination of care between HCPs and health care facilities, ensuring efficient patient follow-up at every stage of the patient’s care pathway.

The platform automatically sends notifications to the various HCPs. These notifications are used, namely, to inform doctors, nurses, and other members of the care team of updates, changes in treatment, or any patient-related event. All platform users have access to real-time information.

The platform allows users to delete a document while the step is in draft mode. Once a step has been submitted, documents can no longer be deleted, as they are sent to the MUHC patient record (Figure 4).

The platform also allows users to add documents in the Documents and Examinations section (Figure 5) at any point along the pathways.

Thus, the platform facilitates communication and sharing of clinical information between health care facilities and between the HCPs involved in the care pathways. Given the sensitive nature of patient data, the platform guarantees data security and confidentiality in compliance with Quebec MSSS regulations on the protection of medical data.

#### Addition of Functionalities Following the Rollout of the Platform

It is important to note that functionalities that were added to the platform were not initially included as part of this pilot project. The aim of these additions was to provide the best, most value-added functionalities to improve, optimize, and automate care pathways workflows to meet the current and future needs of HCPs.

All additions were analyzed, prioritized, developed, tested, and validated with stakeholders. To complete the work, we held several meetings with the platform’s end users to obtain their feedback in an iterative fashion and better identify areas for improvement.

A total of 9 functionalities were added following the results of the survey (objective 3) and consultations with HCPs after the platform’s rollout. These functionalities have been grouped into 5 categories (opening a new request, consultation step, closing the request, administrative notes, and dashboard indicator; Textbox 3).

**Functionalities added following the survey results grouped in 5 categories.**

**Opening a new request**

1. Enable surgeons to initiate a request on the platform themselves. Nurses are usually the ones who initiate requests when a new patient has a scheduled appointment with a surgeon. However, some patients who come in for a follow-up appointment may require surgery. Thanks to this new functionality, the surgeon is no longer dependent on a nurse to create the request for a patient they are seeing for a follow-up visit.

**Consultation step**

2. Enable the nurse and surgeon to change the site of the preoperative visit at the consultation stage. In fact, there were frequent errors when completing this field, which could no longer be modified once the consultation step had been submitted. Correction by the technology partner (Akinox) was then required, resulting in delays and costs. This new functionality gives clinicians full autonomy in the event of an error.

3. Make the selection of a statement mandatory in the transfusion consent form. This ensures that the form is completed and stored in the patient’s file.

4. Include the name of the surgeon who will perform the surgery in the consent form. When the platform was rolled out, the name of every surgeon appeared in the document. For legal reasons, only the name of the surgeon who meets with the patient should appear. The new functionality corrects this problem.

5. Enable patients to sign forms electronically. When the platform was rolled out, surgeons had to print out 3 forms (surgery admission request, informed consent form, and transfusion consent form) for patients to sign before the nurse scanned them and uploaded them to the platform. This new functionality enables patients to sign documents directly on the platform, using a tablet and stylus. The documents are then automatically stored in the patient’s file at the McGill University Health Centre.

6. Enable surgeons to enter their consultation notes before, during, or after the electronic forms are completed. When the platform was rolled out, having to complete a note before completing the forms did not fit well into the workflow of the surgeons, who would normally complete the note when the patient left the room. This functionality gives surgeons greater latitude.

**Closing a request**

7. lose a request on the platform without having the 3 patient discharge documents (discharge summary, operation report, and pathology report). These documents are not actually completed for all patients. The addition of this functionality enables the user to close a request even when one or more documents are missing so the platform dashboard reflects the actual status of these requests.

**Administrative notes**

8. Add administrative notes. The notes are integrated throughout the patient’s care pathways, making them available and visible to all users. They are used to enter information to facilitate patient care management (eg, waiting for an examination before completing the preoperative visit). These notes reduce the need for email or telephone exchanges, making patient care management more efficient.

**Dashboard indicator**

9. Add the clinical priority to indicators. This addition makes it possible to compare surgery-related delays with the clinical priority initially indicated by the surgeon during the consultation.

Several of these functionalities required discussion and validation with various departments, including information security teams, legal affairs, and medical records.

### Objective 3: Implement the Platform and Evaluate the End-User Experience

#### Overview

Postimplementation data were available for extraction from the platform from January 21, 2021, to September 7, 2021. During this period, 106 patients were candidates for thoracic surgery. Of these, 70.8% (75/106) had oncological conditions, and 29.2% (31/106) had nononcological conditions. Of the 106 patient candidates, 58 (54.7%) completed the surgery journey. Among the patient candidates, 75 (70.8%) had an oncological condition and either an ongoing or completed care pathway.

#### Demographic Information

With regard to objective 3 of the study, every participant of the 13 participants completed the survey: 5 (38%) MUHC surgeons, 1 (8%) nurse navigator, and 2 (15%) nurses from the CISSSO, 1 (8%) MUHC nurse navigator, 2 (15%) MUHC nurses, 1 (8%) MUHC central operating room booking medical secretary, and 1 (8%) MUHC thoracic surgery clinic medical secretary.

#### Perceived Benefits of the Platform

All (13/13, 100%) participants either agreed or strongly agreed with the perceived benefits of the platform (Table 1).

**Table 1:** Perceived benefits of the platform (N=13).

Components	Agree, n (%)	Strongly agree, n (%)
Improves the continuity of care and services	8 (62)	5 (38)
Contributes to better demand management and patient intake within the prescribed timeframe	10 (77)	3 (23)
Contributes to the fluidity of the processes	10 (77)	3 (23)
Improves the experience of the platform user	7 (54)	6 (46)
Is user-friendly	7 (54)	6 (46)
Is integrated into my clinical workflow without difficulty	8 (62)	5 (38)
Improves interestablishment coordination of the corridors of care and specialized health services	4 (31)	9 (69)
Ensures the patient’s transition from one establishment to another	6 (46)	7 (54)
Improves communication between health care professionals	7 (54)	6 (46)
Improves the transmission and management of information between health care providers	5 (38)	8 (62)
Rends information available to all users at all times	3 (23)	10 (77)
Improves access to clinical documents required for the service	5 (38)	8 (62)

#### Evaluation of the Platform in the Context of Specific Workflows for Each User Profile

Tables 2-6 show the evaluation of the platform’s effectiveness in the context of workflows by health care facilities and by the HCPs involved in the care pathways. The platform was customized to meet the specific needs of different user profiles. Therefore, each user’s access to the platform’s functionalities was tailored to their role and responsibilities in the patient’s care pathway. The evaluation helped validate whether the clinical and administrative flows specific to each stage of the thoracic surgery care pathways were well supported by the platform.

**Table 2:** Triage, consultation, and preoperative visit—Centre intégré de santé et de services sociaux de l’Outaouais (CISSSO) nurses and nurse navigator (n=3).

Components	Agree, n (%)	Strongly agree, n (%)
The platform facilitates the reception of the request and the triage	2 (67)	1 (33)
The platform ensures the follow-up	2 (67)	1 (33)
The platform facilitates the transmission of clinical documents required for the consultation in thoracic surgery to the patient’s file	0 (0)	3 (100)
The platform facilitates the surgical referral to the MUHC^a^	0 (0)	3 (100)
The platform facilitates referrals for surgery	1 (33)	2 (67)
The platform facilitates the transmission of information relating to the preoperative visit to the CISSSO	0 (0)	3 (100)
The platform facilitates the transmission of information relating to the preoperative visit to the MUHC	0 (0)	3 (100)
The platform allows you to have a notification for the patient’s discharge	3 (100)	0 (0)

^a^MUHC: McGill University Health Centre.

**Table 3:** Preoperative visit and surgery—McGill University Health Centre (MUHC) nurse navigator (n=1).

Components	Agree, n (%)
The platform facilitates referral for surgery at the MUHC	1 (100)
The platform allows you to receive notification that the preoperative is complete and the patient is ready for surgery	1 (100)
The platform facilitates smoother coordination with the operating room	1 (100)
The platform facilitates patient discharge and referral	1 (100)

**Table 4:** Preoperative and surgery—McGill University Health Centre (MUHC) central operating room booking medical secretary (n=1).

Components	Agree, n (%)	Disagree, n (%)
The platform facilitates the opening of the patient record at the MUHC	1 (100)	0 (0)
The platform facilitates referral for surgery at the MUHC	1 (100)	0 (0)
The platform facilitates the data gathering for the patient registration on the surgical waiting list	1 (100)	0 (0)
The platform allows to receive notification that the preoperation at the CISSSO^a^ is complete and the patient is ready for surgery	1 (100)	0 (0)
The platform allows the registration and the change of surgery date	1 (100)	0 (0)
The platform allows you to register the date of the preoperative telephone training at the MUHC	1 (100)	0 (0)
The platform facilitates the preoperative visit to the MUHC	0 (0)	1 (100)

^a^CISSSO: Centre intégré de santé et de services sociaux de l’Outaouais.

**Table 5:** Preoperative and surgery—McGill University Health Centre (MUHC) thoracic surgery medical secretary (n=1).

Components	Strongly agree, n (%)
The platform facilitates referral to surgery at the MUHC	1 (100)
The platform allows you to receive notification that the preoperative is complete and the patient is ready for surgery	1 (100)
The platform allows you to receive notifications reminding you to attach the patient’s postdischarge documents	1 (100)
The platform facilitates the sharing of postdischarge patient documents: discharge summary, operative report, and pathology report	1 (100)

**Table 6:** Consultation—Surgeons or McGill University Health Centre (MUHC; n=5).

Components	Neither, n (%)	Agree, n (%)	Strongly agree, n (%)
The platform facilitates the creation of consultation note	4 (80)	1 (20)	0 (0)
The platform facilitates the referral and follow-up of patients for surgery	0 (0)	4 (80)	1 (20)
The platform keeps track of the referral of patients to oncology, to another specialist, needing follow-up before a decision is made, or not needing surgery	0 (0)	2 (40)	3 (60)
The platform facilitates the electronic signature of forms	2 (40)	2 (40)	1 (20)
The platform facilitates and secures the transmission of information to the MUHC	0 (0)	2 (40)	3 (60)
The platform reduces the risk of loss of information	0 (0)	2 (40)	3 (60)

Feedback from two nurses at the MUHC highlighted unanimous agreement on two key aspects of the platform. Both nurses (2/2, 100%) agreed that the platform facilitates patient discharge and referral. Similarly, both nurses (2/2, 100%) confirmed that the platform effectively facilitates the transmission of information related to the discharge process.

The results indicated that for the initial triage, consultation, and preoperative visit stages, CISSSO nurses and the nurse navigator “agreed” or “strongly agreed” that the platform supports each workflow (Table 2).

The results of the MUHC nurses’ and nurse navigator’s surveys were similarly positive with regard to the platform at specific stages of the care pathways (Table 3).

The MUHC preoperative and surgery secretary and the MUHC thoracic surgery medical secretary “strongly agreed” (Table 5) that the platform is fit for purpose for all flows. That said, the MUHC central operating room booking medical secretary “agreed” that the platform is task-ready, except for the MUHC preoperative visit, where she “strongly disagreed” (Table 4). A functionality (2-consultation step; Textbox 3) has been added to better meet her needs at this specific stage of the care pathways.

The results from surgeons ranged from “neutral” to “strongly agree.” Their feedback led to further adjustments to optimize the platform and ensure that it meets surgeons’ requirements for consultations throughout the care pathways, from triage to patient discharge. In total, 6 functionalities were added after the platform was rolled out (Textbox 3).

#### Evaluation of the Overall End-User Experience

##### Overview

Part 4 of the survey included open-ended questions enabling end users to evaluate their overall experience of the platform during the pilot period. The open-ended questions focused on ease of use, user-friendliness, expected effort, expected performance, perceived usefulness, etc.

##### Perceived Ease of Use

All (13/13, 100%) the HCPs found the platform easy and pleasant to use. Menus and functionalities were organized logically, enabling them to quickly find what they need. Moreover, data were presented in a structured way, enabling HCPs to interpret it easily and make informed decisions based on the patient’s needs. One participant stated the following:

Navigating with the metro line is intuitive and reduces the time spent searching for patient information.

Nurse navigator

Another participant stated the following:

The quality of the platform makes it easy to use as part of our operational reality.

Nurse

All (13/13, 100%) participants stated that the platform is not complicated and requires a minimum of learning time. One participant stated the following:

The platform is easy to use for a dinosaur like me...The software is not slow and is pleasant to use. It’s simple. The visuals are well done.

Surgeon

##### Perceived Usefulness

###### Improving Efficiency

All (13/13, 100%) participants appreciated the easy access to patient information. They can search and download data easily, which saves time and simplifies their tasks. One participant stated the following:

Accessing data is easy, so I can search and download information easily.

Nurse

Another participant mentioned the following:

Ease of access to documents and the speed of sending them.

Medical secretary

Participants reported that medical information is stored securely on the platform, preventing the loss of physical documents. This helps to ensure data confidentiality and integrity. In addition, MUHC surgeons can easily access documents signed at the CISSSO, which eliminates delays in the document treatment process, enabling faster patient care management. One participant stated the following:

There is less risk of losing information and documents between Gatineau and Montreal. Easier access to documents signed in Gatineau avoids delays in processing information. There is less of a need to consult patients’ physical files in Montreal, easier access to all documents, including examination reports such as CT and PET scans, as well as analyses that are performed in Gatineau for Montreal surgeons and anesthetists. This means I can get the information I need quickly, without any delays in transferring documents.

Surgeon

###### Improving Effectiveness

With easy access to data, HCPs can avoid searching for information in different physical files and care settings, enabling them to focus more on patient care.

One participant stated the following:

Information is easy to find. Compared to the emails we used to send...The different users can access it in real time.

Surgeon

Participants reported that they can track a patient’s progress over time, even as they are transferred from one care setting or health care facility to another. Thus, the platform facilitates the exchange of information and continuity of care. This ensures more efficient and secure patient care management throughout the care pathways, facilitating better information and coordination for all care teams, whether it is between the different HCPs or between the CISSSO and the MUHC. The participant stated the following:

The platform facilitates communication and information sharing between care teams and between the MUHC and the CISSSO. We can exchange important information to ensure that care management is adapted to each of our patients.

Nurse navigator

Participants stressed the importance of having complete, up-to-date information on the patient’s health status to make informed decisions regarding diagnosis, treatment, and overall care management. Real-time access to data enables better coordination between the various HCPs involved in the care pathways. The participant stated the following:

Continuous access to patient data reduces risks, improves the quality of care and optimizes health outcomes.

Nurse

Another participant stated as follows:

The solution has facilitated access to patient file numbers, it enables the retrieval of medical notes and avoids duplication of work.

Medical secretary

All (13/13, 100%) participants described how the coordination of transfers between health care facilities becomes more efficient when they have access to the patient’s medical information at every stage of the care pathways. This reduces the risk of losing patients in the health care system, as the information is available to all HCPs involved in the patient’s follow-up. One participant stated the following:

We no longer lose patients in the system.

Surgeon

Another participant stated the following:

By avoiding the loss of patients in the health care system, we also avoid redundant tests and unnecessary medical procedures.

Surgeon

The platform gathers all of a patient’s medical data in one place and enables ongoing monitoring of the patient. One participant stated the following:

The care process is more reliable with the platform, and the platform’s metro line makes it possible to monitor the various steps of the patient’s care pathway.

Surgeon

Most (10/13, 77%) participants mentioned that the platform fits into existing work processes, as it has been designed to offer functionalities that are specific to each user’s role and responsibilities. The platform makes their work easier and more efficient. One participant stated the following:

The platform has a positive impact on our performance because it is compatible and integrates well with aspects of our work and the way we work.

Surgeon

Another participant stated the following:

The platform accurately delivers information and does so in an easy-to-interpret format in which we can perform our tasks when we need to. This allows me to spend more time with my patients.

Nurse navigator

Most (10/13, 77%) participants mentioned that the automation has streamlined workflows, eliminating redundant steps and unnecessary delays. This has led to better time management and more efficient use of HCPs.

##### Facilitating Conditions

Facilitating conditions play an important role in the successful adoption and optimal use of the platform. Users had ongoing technical support to help them with any platform-related technical issues. This technical support reduced the frustration associated with technical issues and encouraged them to continue using it with confidence. Two participants stated the following:

I feel supported and confident in using the platform.

Nurse

Someone is always available to help me with any platform-related issues.

Nurse navigator

Participants emphasized that they received individual training to learn the platform’s functionalities and that they had a clear, detailed user guide at their disposal.

## Discussion

### Principal Findings

Our research has shown that the thoracic surgery care pathways are complex and require effective coordination of interfacility service corridors (objective 1). The oncology and nononcology care pathways are not limited to the surgical procedure itself. Pathways also include triage, preoperative preparation, postoperative follow-up, rehabilitation, and overall patient care management throughout the care pathway. This continuity of care requires seamless communication between the different HCPs and health care facilities. The main challenge is that the 2 health care facilities operate in silos. When the MUHC and the CISSSO operate in isolation, without effective communication and information sharing, it can lead to problems at transition points in the care pathways. It can also impede continuity of care, leading to duplication, errors, or delays in treatment and follow-up, with potential consequences for patients’ health and quality of life. However, the implementation of a digital health solution can play a role in the coordination and efficiency of health care, leading to integrated, coordinated, and equitable patient care at every step of the care pathways.

The information gathered in objective 1 provided an understanding of the entire patient journey and the activities in which HCPs are involved. Mapping the care pathways using the Business Process Model Notation method provided precise indications of the user interface, system integrations, functionalities, workflows, and dataflows required to optimize the interfacility thoracic surgery care pathways. Analysis of this mapping helped pinpoint the steps that do not add value for HCPs or are missing as well as any opportunities for improvement, aiming to better meet their clinical and administrative needs.

Our research also demonstrated that using the participatory design approach from the outset of the platform design process in conjunction with future users and other key stakeholders was beneficial (objective 2). The workshops enabled participants to share their needs, expectations, and priorities regarding platform functionalities. These exchanges contributed to a better understanding of specific use cases and issues facing future users. Involving them as stakeholders in the design process strengthened their sense of ownership and commitment to the developed solution. The knowledge generated through the workshops was used to enhance the prototype under development. Thus, by integrating coproduced knowledge, we were able to ensure that the platform corresponded to the actual needs and expectations of future users, increasing the chances of adoption and acceptance of the solution. This user-centered approach is conducive to creating a design that is better suited to the real needs of end users and contributes to a better overall user experience.

The survey results (objective 3) showed that all HCPs “agreed” or “strongly agreed” on the benefits of the platform. For the vast majority, the clinical and administrative flows for each user profile are well supported by the platform. To ensure continuous improvement of the platform, 9 functionalities were added in response to end-user feedback, representing significant added value to the care pathways.

Evaluation of the end-user experience (objective 3) demonstrated several benefits resulting from the platform. First, the thoracic surgery care pathways have been optimized, automated, and made more secure. This ensures better connectivity between the different players, facilitating the flow of exchanges and information traceability, while ensuring that each player has a better understanding of the patient care process at the MUHC and the CISSSO.

Second, the platform has helped overcome the challenges associated with operating in silos. It facilitates communication between HCPs and between the MUHC and the CISSSO, which can lead to better decision-making for patients. In turn, this reduces the risk of medical errors and improves efficiency by optimizing case processing time and improving information transfer. These improvements have made it possible to reallocate staff at both health care facilities from searching for and monitoring information to activities that add value to the care pathways.

Therefore, workflows are more efficient and effective, and resources are better used. This was enabled at both health care facilities by eliminating unnecessary or duplicated steps, reducing delays in the process, improving the fluidity of the care pathways, giving all HCPs access to information related to completed and upcoming care pathway steps, and providing access to performance indicators across the entire care pathways with a view to continuous improvement for the benefit of patients.

In addition, the platform promotes better care coordination by streamlining transitions between care settings across the entire health care continuum. This enhanced coordination ensures that patients benefit from comprehensive, coherent care throughout their care pathways.

Finally, the success of the project convinced the clinical teams and senior management of the health care facilities (MUHC and CISSSO) to pursue the long-term use of the Akinox digital health platform for the oncology and nononcology thoracic surgery care pathways.

### Limitations and Future Research

While the study represents a significant advancement in the field of thoracic surgery care pathways and interfacility health care service coordination, there are some limitations that need to be addressed to ensure the generalizability, sustainability, and overall effectiveness of the integrated digital health solution.

One limitation is the lack of validation of the platform’s effectiveness across different health care contexts and specialties. Further research is needed to assess its generalizability across varied health care environments and specialties beyond thoracic surgery and develop evidence-based guidelines for implementation in diverse clinical contexts.

In addition, the study lacks a comprehensive assessment of patients’ experiences and satisfaction with the digital health solution. Understanding patients’ perspectives is important for evaluating the overall effectiveness and impact of the integrated digital health platform on patient-centered care. Future research should include patient feedback to enrich our understanding of how the platform influences patient outcomes and experiences.

Furthermore, the quantitative evaluation of the user experience is based on a limited sample of 13 users. Although these initial findings provide valuable insights, the small sample size reduces the ability to generalize the results to a broader population. Expanding the user base in future studies is essential to capturing a more diverse range of experiences and ensuring the platform’s adaptability and effectiveness across different user groups.

Finally, for future research, it would be valuable to statistically measure the process over time to assess the real improvements following the platform’s implementation. This analysis could offer deeper insights into the platform’s impact on operational efficiency and patient outcomes, providing data that can inform continuous improvement efforts.

### Conclusions

This pilot project helped develop a usable and valid target care pathway model for the interfacility thoracic surgery care pathways by integrating a platform. The platform’s infrastructure is designed to be easily configurable and adaptable to different types of cancer and surgery. This means that the model can be extended to other medical specialties, enabling a smoother care pathway for a greater number of patients.

The platform also provides a technological solution and model that can be exported to the pulmonary oncology network and other care networks and clinical units. Furthermore, this pilot project highlights the best practices and conditions for success in the consolidation of a cancer network that can be transferred to other networks, namely the key role played by nurse navigators, who are the guarantors of the patient’s care pathway.

Our pilot project is in line with one of the objectives of the MSSS’s Information Technology Division, which aims to use information resources in the health care network to make the shift to digital technology by improving business processes. This project is part of Quebec’s Digital Strategy, launched by the Ministère de l’Économie et de l’Innovation: one of its orientations is to have connected health care for the citizens. The Ministère de l’Économie et de l’Innovation believes that digital technology makes it possible to respond to patients’ needs according to their realities, optimizing and improving health care services. Collaboration and sharing, in this case, between HCPs from different health care facilities and even with patients, represent the future of the integrated, patient-centered health care system.

In terms of managerial insights, the study highlights the importance of strategic leadership in the implementation of digital health solutions. By fostering collaboration between different stakeholders, organizations can improve care coordination and operational effectiveness. This is in line with health care that emphasizes the need for patient-centered care approaches.

In addition, this entire pilot project is part of the MSSS’s approach, aiming to improve the accessibility, equity, integration, and quality of services and care. This model, which is increasingly patient-centered, should help provide care and services within medically acceptable timeframes; it should be transferable to other care pathways and health care facilities and contribute, in this case, to better care and services for patients with cancer.

Finally, this study pushes the boundaries of theoretical advancements in medical informatics by bridging the gap between digital solutions and practical applications in clinical settings. It emphasizes the role of technology not just as a tool but as an integral part of a patient’s care pathway, thereby enhancing the theoretical frameworks on health informatics.

## Data Availability

The datasets generated during and analyzed during this study are available from the corresponding author on reasonable request.

## Appendix

Multimedia Appendix 1 COREQ (Consolidated Criteria for Reporting Qualitative Research) checklist.

## Abbreviations

CISSSO: Centre intégré de santé et de services sociaux de l’Outaouais
COREQ: Consolidated Criteria for Reporting Qualitative Research
HCP: health care provider
ICT: information and communication technology
IFSR: interfacility service request
LCSC: local community service center
MSSS: Ministère de la Santé et des Services sociaux
MUHC: McGill University Health Centre
