# Supplementary material for: Enhancing Care Coordination in Oncology and Nononcology Thoracic Surgery Care Pathways Through a Digital Health Solution: Mixed Methods Study
Source: JMIR Form Res. 2024 Nov 26;8:e60222. doi: 10.2196/60222 (PMC11632290; doi:10.2196/60222)
Supplement: Multimedia Appendix 1 [file formative_v8i1e60222_app1.pdf]

Multimedia Appendix I: Consolidated criteria for reporting qualitative studies (COREQ): 32-item checklist

No. Item	Guide questions/description	Reported on Page #
**Domain 1: Research team and reflexivity**		
Personal Characteristics		
1. Interviewer/facilitator	Which author/s conducted the interview or focus group?	Title Page
2. Credentials	What were the researcher's credentials? E.g. PhD, MD	Title Page
3. Occupation	What was their occupation at the time of the study?	Title Page
4. Gender	Was the researcher male or female?	Title Page
5. Experience and training	What experience or training did the researcher have?	Title Page
Relationship with participants		
6. Relationship established	Was a relationship established prior to study commencement?	Page 5
7. Participant knowledge of the interviewer	What did the participants know about the researcher? e.g. personal goals, reasons for doing the research	Participants did not know researchers' goals
8. Interviewer characteristics	What characteristics were reported about the interviewer/facilitator? e.g. Bias, assumptions, reasons and interests in the research topic	No characteristics were reported regarding the interviewer/researcher
**Domain 2: study design**		
Theoretical framework		
9. Methodological orientation and Theory	What methodological orientation was stated to underpin the study? e.g. grounded theory, discourse analysis, ethnography, phenomenology, content analysis	Abstract, Pages 5, 6 and 7
Participant selection		
10. Sampling	How were participants selected? e.g. purposive, convenience, consecutive, snowball	Pages 5, 6 and 7
11. Method of approach	How were participants approached? e.g. face-to-face, telephone, mail, email	Pages 5, 6 and 7
12. Sample size	How many participants were in the study?	Abstract, Pages 5, 6 and 7
13. Non-participation	How many people refused to participate or dropped out? Reasons?	All participants agreed to take part in the study. Pages 6 and 7
Setting		
14. Setting of data collection	Where was the data collected? e.g. home, clinic, workplace	Pages 6 and 7 .
15. Presence of non-participants	Was anyone else present besides the participants and researchers?	Pages 6 and 7

16. Description of sample	What are the important characteristics of the sample? e.g. demographic data, date	Pages 5, 6 and 7
Data collection		
17. Interview guide	Were questions, prompts, guides provided by the authors? Was it pilot tested?	Pages 5, 6 and 7
18. Repeat interviews	Were repeat interviews carried out? If yes, how many?	No
19. Audio/visual recording	Did the research use audio or visual recording to collect the data?	Pages 5 and 6
20. Field notes	Were field notes made during and/or after the interview or focus group?	Yes, Page 6
21. Duration	What was the duration of the interviews or focus group?	Page 5
22. Data saturation	Was data saturation discussed?	A total of 27 interviews were conducted, which provided a comprehensive range of insights
23. Transcripts returned	Were transcripts returned to participants for comment and/or correction?	Page 5
**Domain 3: analysis and findings**		
Data analysis		
24. Number of data coders	How many data coders coded the data?	N/A
25. Description of the coding tree	Did authors provide a description of the coding tree?	N/A
26. Derivation of themes	Were themes identified in advance or derived from the data?	Pages 5 and 6
27. Software	What software, if applicable, was used to manage the data?	Pages 5 and 6
28. Participant checking	Did participants provide feedback on the findings?	No
Reporting		
29. Quotations presented	Were participant quotations presented to illustrate the themes/findings? Was each quotation identified? e.g. participant number	Pages 25-28
30. Data and findings consistent	Was there consistency between the data presented and the findings?	Yes, Pages 7-28
31. Clarity of major themes	Were major themes clearly presented in the findings?	Yes, Pages 7-28
32. Clarity of minor themes	Is there a description of diverse cases or discussion of minor themes?	Yes, Pages 8-12, 22-28
